# T-Lymphocyte Subsets Alteration, Infection and Renal Outcome in Advanced Chronic Kidney Disease

**DOI:** 10.3389/fmed.2021.742419

**Published:** 2021-09-09

**Authors:** Jiachuan Xiong, Yu Qiao, Zhikai Yu, Yinghui Huang, Ke Yang, Ting He, Jinghong Zhao

**Affiliations:** The Key Laboratory for the Prevention and Treatment of Chronic Kidney Disease of Chongqing, Department of Nephrology, Chongqing Clinical Research Center of Kidney and Urology Diseases, Xinqiao Hospital, Army Medical University (Third Military Medical University), Chongqing, China

**Keywords:** T-lymphocyte, infection, renal outcome, chronic kidney disease, risk factors

## Abstract

**Background:** T-lymphocyte subsets reflect patients' immune status and are associated with adverse outcomes in various diseases. However, the association between T-lymphocyte subsets and major infection and renal outcome in chronic kidney disease (CKD) patients has not been well-addressed.

**Methods:** Patients diagnosed with stage 3–5 of non-dialysis CKD were recruited, and healthy subjects were selected as the controls. T-lymphocyte subsets (CD3^+^, CD4^+^, CD8^+^) were detected by flow cytometry, and the CD4^+^/CD8^+^ T cell ratio was then calculated. Patients were divided into the normal-level group and the low-level group according to the clinical reference value. The primary outcomes were the major infection and renal outcome.

**Results:** A total of 410 CKD patients were enrolled; the average age was 47.25 years. Compared to the healthy controls, the level of CD3^+^, CD4^+^, CD8^+^ T cells, and the CD4^+^/CD8^+^ T cell ratio were significantly decreased in CKD patients (*p* < 0.05). During the median follow-up of 2.56 (quartile interval 1.24–3.46) years, major infections occurred in 15.10% of the CKD patients. The incidence of infection was significantly higher in the low-level group of CD3^+^, CD4^+^ T cells, and CD4^+^/CD8^+^ T cell ratio compared with the normal level groups. Kaplan-Meier analysis showed that the lower level of CD3^+^, CD4^+^ T cells, and CD4^+^/CD8^+^T cell ratio is associated with a greater risk of infection. Cox regression analysis further confirmed that low CD3^+^, CD4^+^ T cells, and CD4^+^/CD8^+^ T cell ratio were independent risk factors of infection in CKD patients. Moreover, during the follow-up, renal events occurred in 37.50% of patients. Kaplan-Meier analysis indicated that low levels of CD3^+^, CD4^+^, and CD8^+^ T cells are significantly associated with renal outcome in CKD patients. Cox regression analysis showed that low level of CD3^+^ T cells (HR = 2.407, 95% CI: 1.664–3.482, *p* < 0.001), CD4^+^ T cells (HR = 2.397, 95% CI: 1.633–3.518, *p* < 0.001) and CD8^+^ T cells (HR = 2.416, 95% CI: 1.476–3.955, *p* < 0.001) were independent risk factors for renal outcome after multivariable-adjusted.

**Conclusion:** CKD patients had a defect in T-lymphocyte subpopulation. T-lymphocyte subsets were closely associated with infection and renal outcome in CKD patients. Suggesting T-lymphocyte subsets are independent predictors of infection and renal outcome in CKD patients.

## Introduction

Chronic kidney disease (CKD) has become a global public health problem, resulting in a heavy burden for all countries (1, 2). Patients will eventually develop end-stage kidney disease (ESKD) due to the irreversible deterioration of kidney function, which requires lifelong replacement therapy. Consequently, it seriously influences the quality of life and overall survival (3). Abnormal immune function, especially cell immunodeficiency, plays a vital role in the progression of CKD (4, 5). This phenomenon not only results in the occurrence of CKD but also aggravates metabolic disorders, oxidative stress, and toxin accumulation in CKD milieus (6, 7). In addition to cellular immunodeficiency and imbalanced immunity, humoral immunity is further suppressed (8). The vicious cycle between cellular immunodeficiency and CKD ultimately becomes an essential driver of ESKD and dialysis outcomes (9).

T-lymphocyte is the most critical cell in cellular immunity and participates in almost all specific immune responses of the body (10). A previous study showed a significant reduction in T-lymphocyte numbers in CKD patients (11). Another study showed that T-lymphocytes in children with CKD without dialysis were less than those in healthy people and decreased gradually as the disease progressed (12). Moreover, T-lymphocytes were also dysregulated in dialysis patients (13). Some kidney diseases, such as lupus nephritis, were reported to have a reduction of peripheral blood CD4^+^T cells and CD4^+^/CD8^+^ T cell ratio compared with that of healthy individuals (14, 15). Moreover, T-lymphocyte is also reported to play a decisive role in early kidney damage in diabetic nephropathy (DN) and promote the development of DN (16). Changes in peripheral blood T-lymphocyte subsets in type 2 diabetes were manifested as decreased CD3^+^ T cells, CD4^+^ T cells, and CD4^+^/CD8^+^ T cell ratio and increased or decreased CD8^+^ T cells (17). Besides, immunosuppressive therapy for some kidney diseases leads to T-lymphocyte numbers drop in pre-dialysis CKD patients (18, 19).

Peripheral blood T cells subsets (including CD3^+^, CD4^+^, CD8^+^ and CD4^+^/CD8^+^) are clinical indicators of cellular immune function. Generally, healthy people have a CD4^+^/CD8^+^ T cell ratio of 1, while changing in this value increases morbidity and mortality (20, 21). As a clinical indicator of cellular immune function, the relationship between T-lymphocyte subsets and CKD progression deserves further study. However, few studies have comprehensively evaluated the association between peripheral blood T cells subsets and the outcome of infection and renal replacement therapy in pre-dialysis CKD patients. Therefore, we conducted a retrospective study in pre-dialysis CKD stage 3–5 patients to assess whether T-lymphocyte subsets can be used as an indicator of major infection and renal outcome.

## Methods

### Study Population

This retrospective cohort study enrolled pre-dialysis CKD stage 3–5 patients from Xinqiao Hospital of Army Medical University in China from January 2015 to July 2018. A total of 410 individuals performed serum T-lymphocyte subsets detection. The study protocol was approved by the ethical committee of Xinqiao Hospital. Informed consent was obtained from all individual participants included in the study.

### Inclusion and Exclusion Criteria

The inclusion criteria were as follows: (1) Pre-dialysis CKD patients (stages 3–5); (2) adults aged 18 years or older; and (3) completed therapeutic information and follow-up data.

The exclusion criteria were as follows: (1) patients with acute kidney injury; (2) patients with various infections of different etiologies; (3) pregnant women; (4) patients with past or current malignancy; (5) patients with congenital or acquired immunodeficiency.

### Clinical Data Collection and Laboratory Analyses

Flow cytometry enumeration of T-lymphocyte subsets was performed on a FACSCalibur system (BD Biosciences, San Jose, CA, USA), and the percentages of T-lymphocyte subsets were analyzed using multiphase software (BD Biosciences, San Jose, CA). For laboratory measurements, blood was available from each patient early in the morning after an overnight fast. Laboratory data, including serum hemoglobin, albumin, creatinine, uric acid, urea nitrogen, calcium, phosphorus, intact parathyroid hormone (iPTH), and cystatin C, were analyzed by a Beckman AU5800 automatic biochemical analyzer (Beckman Coulter, Inc., Brea, CA) according to the manufacturer's protocol. The estimated glomerular filtration rate (eGFR) was calculated by the CKD Epidemiology Collaboration (CKD-EPI) creatinine equation (22).

### Investigation of Study Outcomes

The renal outcome is defined as receiving renal replacement therapy due to irreversible deterioration of kidney function, including hemodialysis, peritoneal dialysis, or kidney transplantation. And the major infection is defined as an infection which is requiring hospitalization for anti-infection treatment.

### Patients Grouping

CKD patients were divided into two groups according to the value of T-lymphocyte subsets: The T-lymphocyte subsets in the peripheral blood of the patients were measured by direct immunofluorescence staining with flow cytometry and were grouped according to their clinical reference value, with Normal-level group (770≤CD3^+^≤2860; 500≤CD4^+^≤1440; 238≤CD8^+^≤1250; 1.0 ≤CD4^+^/CD8+≤ 2.47) and Low-level group (CD3^+^ <770; CD4^+^ <500; CD8^+^ <238; CD4^+^/CD8^+^ <1).

### Statistical Analysis

Continuous variables with normal distribution were reported as means and standard deviations, and other continuous variables that accord with non-normal distribution were reported as medians (25th, 75th percentiles). Categorical variables were recorded as percentages. The characteristics of the participants were compared by the vital status of T-lymphocyte subsets using independent sample *t*-tests or Wilcoxon rank-sum tests for continuous variables and chi-square tests for categorical variables. Kaplan–Meier curves were plotted, and log-rank tests were used to compare the progression-free survival for renal outcome and infection according to T-lymphocyte subsets. Cox proportional hazards models were used to examine the relationship between CD3^+^, CD4^+^, CD8^+^ T and CD4^+^/CD8^+^ T cell ratio and renal outcome, major infection. The effect of potential confounding was analyzed by constructing models with incremental adjustments: Model 1 (unadjusted model); Model 2 (Model 1 adjusted by age, gender, BMI, systolic blood pressure, and diastolic blood pressure); Model 3 (Model 2 adjusted by CKD stage, hypertension, diabetes, cardiovascular disease, and smoker); Model 4 (Model 3 adjusted by phosphorus, calcium, intact parathyroid hormone, hemoglobin, albumin, BUN, cystatin C, and uric acid). All statistical analyses were carried out using the SAS software package version 9.4 (SAS Institute, Cary, NC, USA), GraphPad Prism software package version 8 (La Jolla, CA, USA), and R-studio software version 3.6.2 (package: forest plot). A *p*-value < 0.05 was considered statistically significant.

## Results

### Alteration of T Lymphocyte Subsets in CKD Population

**Figure 1:**
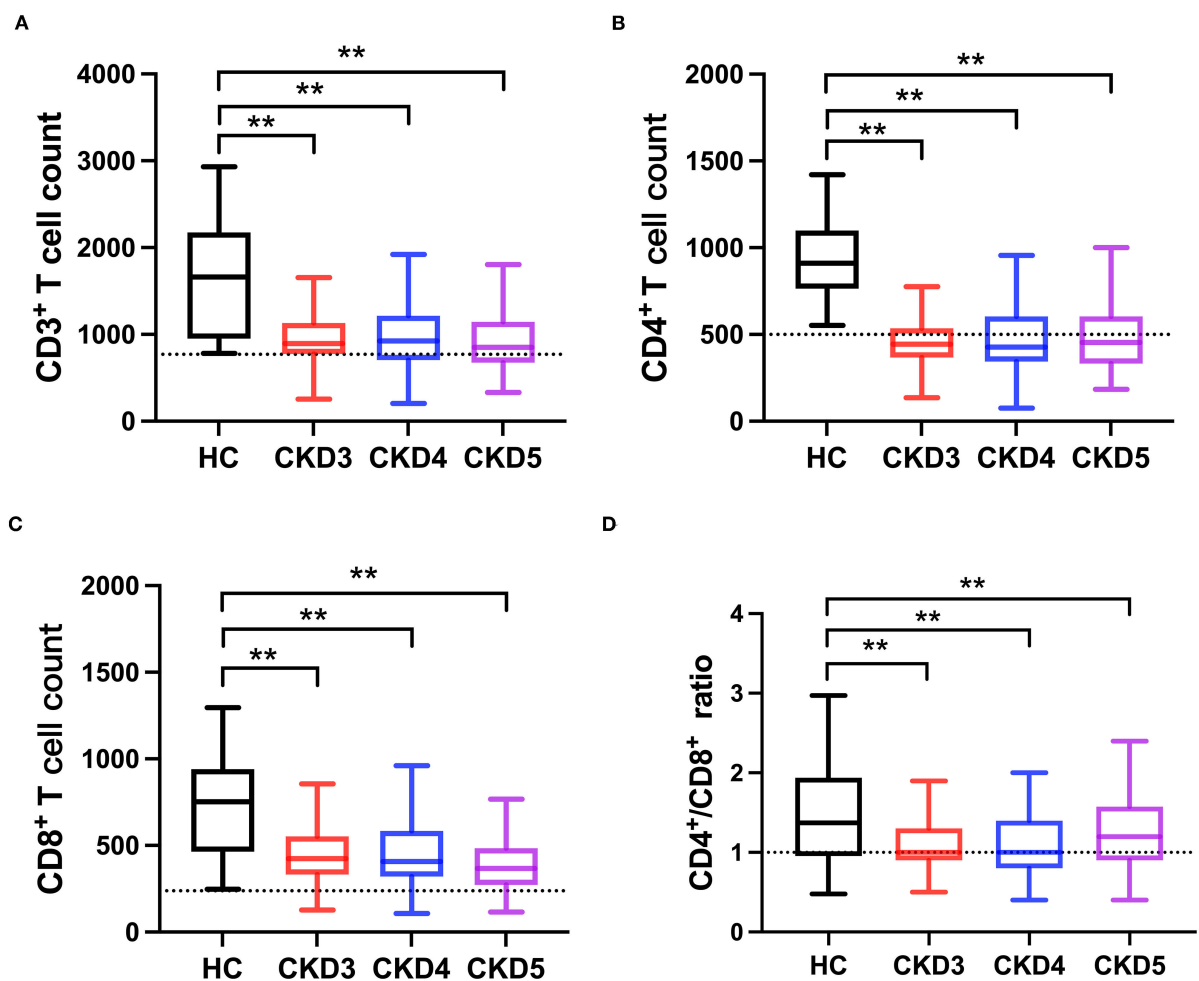
Comparison of T-lymphocyte subsets between CKD patients and healthy subjects (HC). **(A)** CD3^+^ T cell count, **(B)** CD4^+^ T cell count, **(C)** CD8^+^ T cell count, **(D)** CD4^+^/CD8^+^ T cell ratio. The dotted line represented the normal value. **p < 0.01.

A total of 623 CKD patients were screened, and 213 patients were excluded. Finally, 410 CKD stage 3–5 patients were included for analysis, and the flow chart is shown in Supplementary Figure 1. The baseline characteristics of the study population are described in Table 1. The mean age was 47.25 years. Approximately 50% of the patients were female. The median eGFR was 26.90 (interquartile range, 13.71–43.52) mL/min/1.73 m^2^. Moreover, about 27% of the patients were smokers, and the prevalence of diabetes was 9.02%. Patients in CKD stage 4 had the highest incidence of diabetes (*p* < 0.05). Furthermore, serum hemoglobin, calcium, and eGFR were lower in advanced stages of CKD, while systolic blood pressure, phosphorus, uric acid, urea nitrogen, cystatin C, and iPTH concentrations were higher in advanced stages of CKD (Table 1). Then, we used 100 healthy adults subjects who were recruited from the physical examination center without any chronic diseases or drug dependence as control; 51% were males with an average of 45.3 years old. The frequency distribution of CD3^+^, CD4^+^, and CD8^+^ T cell numbers was shown in Supplementary Figure 2. The highest CD4^+^/CD8^+^ T cell ratio was found in stage 5 CKD. Compared with the healthy subjects, the CD3^+^ T cell count in CKD stage 3–5 patients was significantly lower than that in the healthy control group (*p* < 0.01). Similarly, CD4^+^, CD8^+^ T cell counts and CD4^+^/CD8^+^ T cell ratio were significantly lower in CKD patients than healthy subjects (*p* < 0.05), as shown in Supplementary Table 1; Figure 1.

**Table 1:** The basic characteristics of the included CKD patients.

**Characteristics**	**Total (*n* = 410)**	**CKD3 (*n* = 187)**	**CKD4 (*n* = 115)**	**CKD5 (*n* = 108)**	**F/χ2**	***P***
Age, years	47.25 ± 14.56	46.11 ± 14.12	48.06 ± 15.48	48.37 ± 14.27	1.075	0.342
Female, *n* (%)	198 (48.29)	92 (49.20)	43 (37.39)	63 (58.33)	9.895	0.007
Current smoker, *n* (%)	111 (27.03)	49 (26.20)	40 (34.78)	22 (20.37)	5.991	0.050
Hypertension, *n* (%)	165 (40.24)	80 (42.78)	48 (41.74)	37 (34.26)	2.216	0.330
Diabetes, *n* (%)	37 (9.02)	14 (7.49)	16 (13.91)	7 (6.48)	4.737	0.094
Cardiovascular disease, *n* (%)	18 (4.39)	7 (3.74)	7 (6.09)	4 (3.70)	1.096	0.578
Body-mass index, kg/m^2^	23.49 ± 3.56	23.88 ± 3.53	23.46 ± 3.31	22.86 ± 3.80	2.842	0.059
Systolic blood pressure, mmHg	130 (120, 143)	130 (119, 139)	130 (120, 145)	135 (122, 150)	11.259	0.004
Diastolic blood pressure, mmHg	80 (74, 90)	80 (74, 90)	82 (74, 90)	81 (76, 90)	2.063	0.356
Hemoglobin, g/L	108.83 ± 23.86	119.70 ± 20.65	109.37 ± 21.74	89.44 ± 18.59	75.017	<0.001
Albumin, g/L	39.20 (35.00, 42.80)	39.80 (35.55, 43.60)	38.50 (33.98, 42.33)	38.60 (35.15, 42.00)	4.603	0.100
Phosphorus, mmol/L	1.21 (1.04, 1.40)	1.14 (1.00, 1.28)	1.19 (1.04, 1.36)	1.40 (1.23, 1.64)	62.241	<0.001
Calcium, mmol/L	2.20 (2.09, 2.30)	2.23 (2.14, 2.31)	2.17 (2.07, 2.31)	2.17 (2.06, 2.28)	7.926	0.019
iPTH, pg/mL	94.80 (54.70, 199.00)	66.30 (45.05, 90.95)	107.00 (60.20, 159.80)	257.60 (163.68, 457.78)	147.509	<0.001
Creatine, μmol/L	213.00 (144.30, 358.20)	138.10 (120.70, 163.50)	253.55 (212.55, 305.75)	470.50 (396.33, 611.75)	320.838	<0.001
Uric acid, μmol/L	469.25 ± 116.05	433.29 ± 103.04	491.53 ± 118.90	507.11 ± 117.00	18.162	<0.001
BUN, mmol/L	10.61 (7.53, 15.89)	7.47 (6.30, 9.13)	11.31 (9.48, 13.83)	20.70 (15.91, 26.40)	253.115	<0.001
eGFR, mL/min/1.73 m^2^	26.90 (13.71, 43.52)	44.00 (37.38, 52.00)	21.40 (18.00, 26.31)	8.92 (6.20, 11.41)	348.521	<0.001
Cystatin C, mg/L	2.67 (1.79, 4.05)	1.70 (1.42, 2.07)	2.90 (2.43, 3.37)	4.53 (4.03, 5.68)	267.050	<0.001

*CKD, chronic kidney disease; iPTH, intact parathyroid hormone; BUN, blood urea nitrogen; eGFR, estimated glomerular filtration rate*.

### T-Lymphocyte Subsets and Major Infection

**Figure 2:**
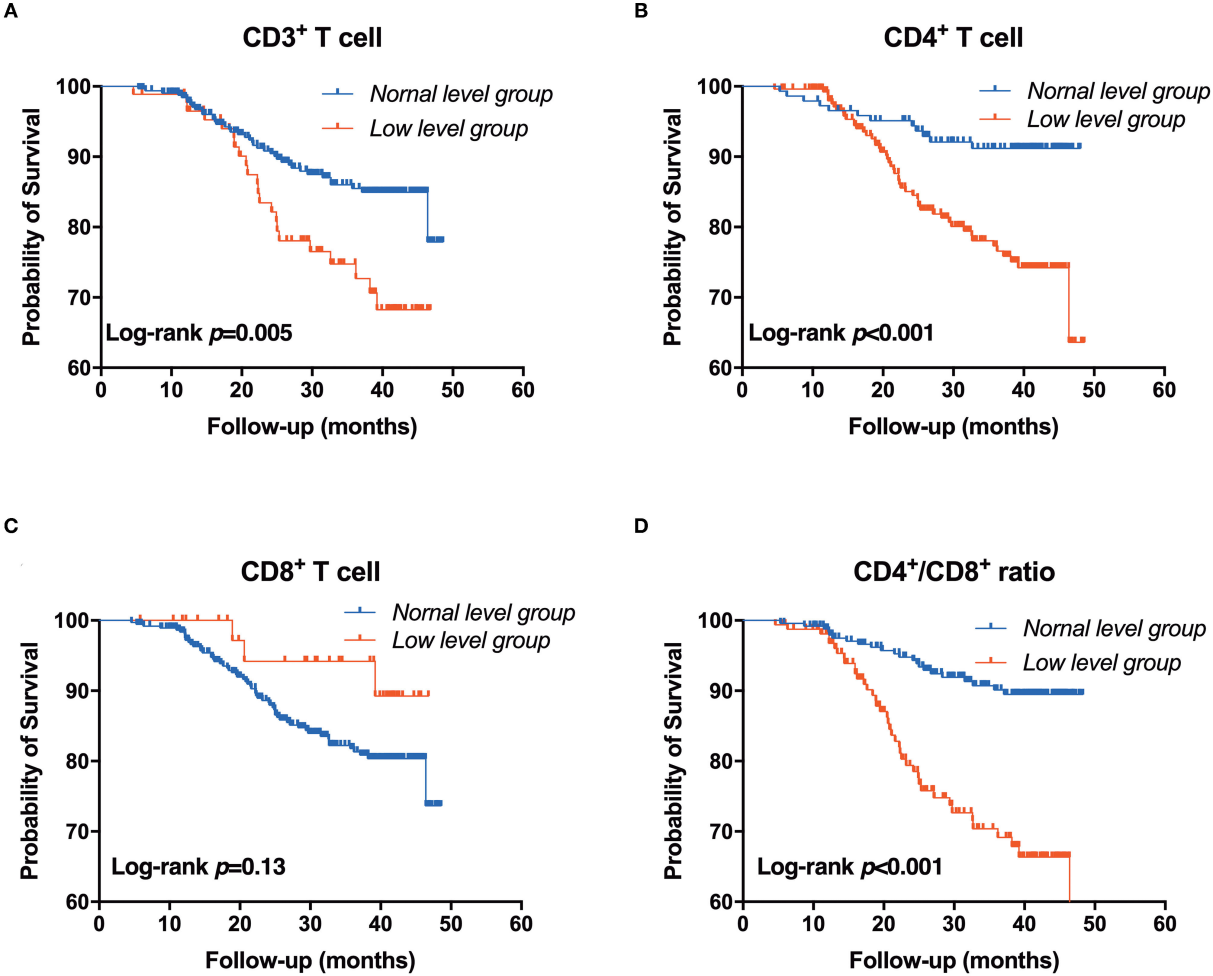
Association between T-lymphocyte subsets and major infection. **(A)** CD3^+^ T cell, **(B)** CD4^+^ T cell, **(C)** CD8^+^ T cell, **(D)** CD4^+^/CD8^+^ T cell. Patients were stratified into two groups by clinical reference value (Kaplan-Meier analysis with log-rank test).

**Figure 3:**
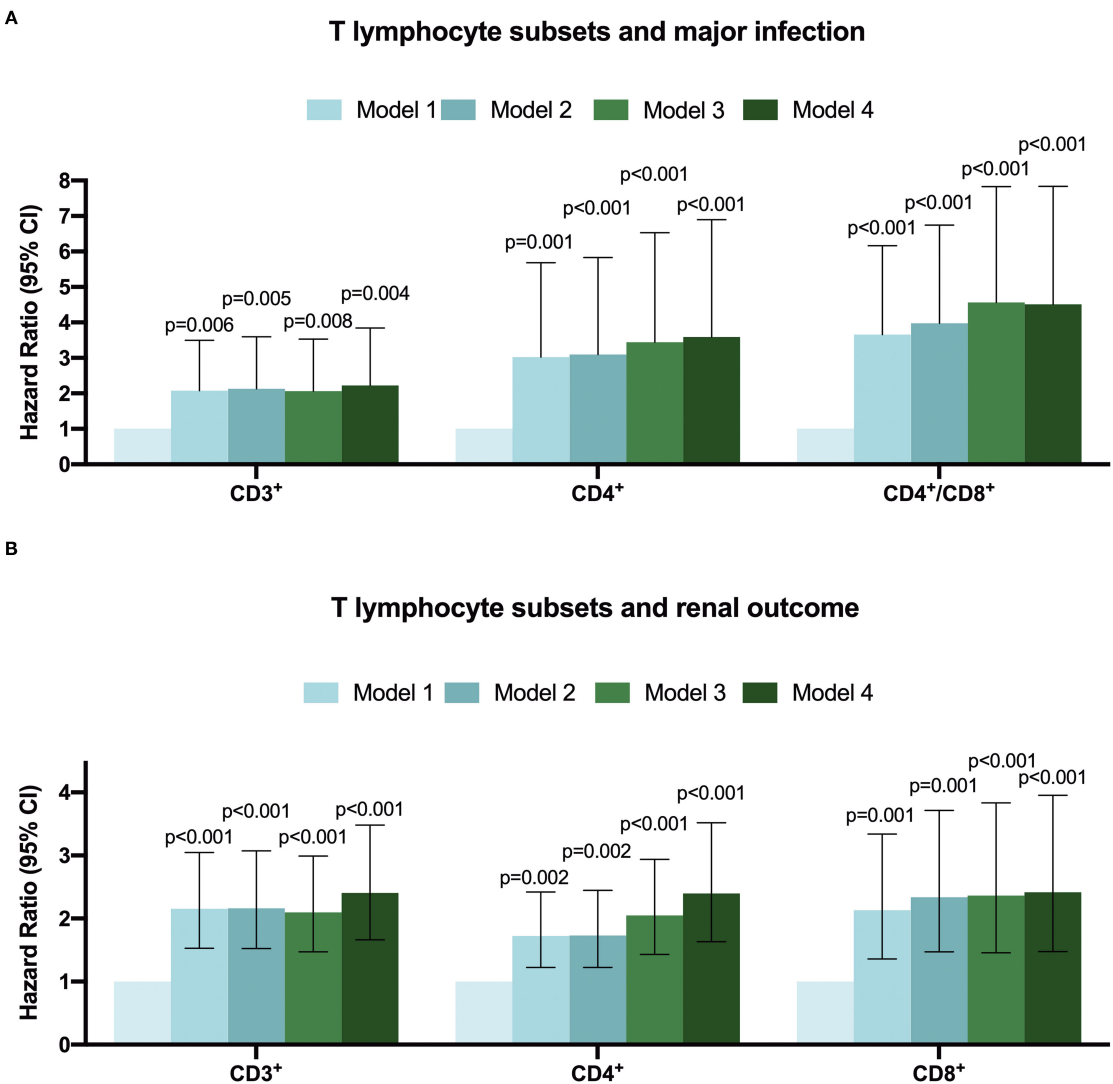
The Hazard ratio for T-lymphocyte subsets with major infection **(A)** renal outcome **(B)** in CKD patients by multivariate-adjusted models.

During a median follow-up of 2.56 (inter-quartile, 1.24–3.46) years, the major infections occurred in 62 (15.12%) cases, of which 27 (43.55%) were female. The sites of infection were respiratory tract infection (pneumonia) in 42 (67.74%) patients and followed by urinary tract infection in 19 (30.65%) patients, and one patient was diagnosed with a blood infection. Then, CKD patients were divided into infection and non-infection groups, as shown in Supplementary Table 2. There was a significant difference between the two groups in CKD stages, serum creatinine, uric acid, BUN and cystatin C, and eGFR. Moreover, compared with the normal level T lymphocyte group, more infection events occurred in the low CD3^+^, CD4^+^ and CD4^+^/CD8^+^ ratio T lymphocyte group except for the low CD8^+^ T lymphocyte group (Supplementary Table 3). The Kaplan-Meier survival analysis was performed to evaluate the relationship between T lymphocyte subsets and the incidence of infection in patients with CKD. Results showed that patients with low levels of CD3^+^, CD4^+^ and CD4^+^/CD8^+^ T cells have a higher risk of major infection when compared with the normal level T lymphocyte group, while there was no significant difference in the infection events between the low level of CD8^+^ T cells and the normal level of CD8^+^ T cells (Figure 2). In addition, Cox regression analysis showed that patients in low CD3^+^ (HR = 2.225, 95% CI: 1.289–3.841, *p* = 0.004), CD4^+^ (HR = 3.591, 95% CI: 1.870–6.896, *p* < 0.001), and CD4^+^/CD8^+^ ratio (HR = 4.507, 95% CI: 2.592–7.837, *p* < 0.001) T lymphocyte group had a greater risk of major infection than patients with normal level T lymphocyte group in multivariable-adjusted models (Table 2; Figure 3A).

**Table 2:** Associations of CD3^+^, CD4^+^, and CD8^+^ T cells with major infection.

	**Model**	**HR (95% CI)**		***P***
		**Low-level group**	**Normal-level group**	
**CD3** ^**+**^				
	Model 1	2.077 (1.233–3.498)	Reference	0.006
	Model 2	2.121 (1.253–3.591)		0.005
	Model 3	2.064 (1.206–3.531)		0.008
	Model 4	2.225 (1.289–3.841)		0.004
**CD4** ^**+**^				
	Model 1	3.024 (1.609–5.683)	Reference	0.001
	Model 2	3.091 (1.641–5.820)		<0.001
	Model 3	3.441 (1.814–6.527)		<0.001
	Model 4	3.591 (1.870–6.896)		<0.001
**CD4** ^**+**^ **/CD8** ^**+**^				
	Model 1	3.658 (2.171–6.166)	Reference	<0.001
	Model 2	3.967 (2.336–6.737)		<0.001
	Model 3	4.562 (2.657–7.832)		<0.001
	Model 4	4.507 (2.592–7.837)		<0.001

*Cox regression model for the association between CD3^+^, CD4^+^, CD8^+^ T cells level, and major infection*.

*Model 1: crude*;

*Model 2: age, gender, BMI, systolic blood pressure and diastolic blood pressure adjusted*;

*Model 3: Model 2 plus CKD stage, hypertension, diabetes, cardiovascular disease and smoker adjusted*;

*Model 4: Model 3 plus phosphorus, calcium, intact parathyroid hormone, hemoglobin, albumin, BUN, cystatin C, and uric acid adjusted*.

### T Lymphocyte Subsets and Renal Outcome

**Figure 4:**
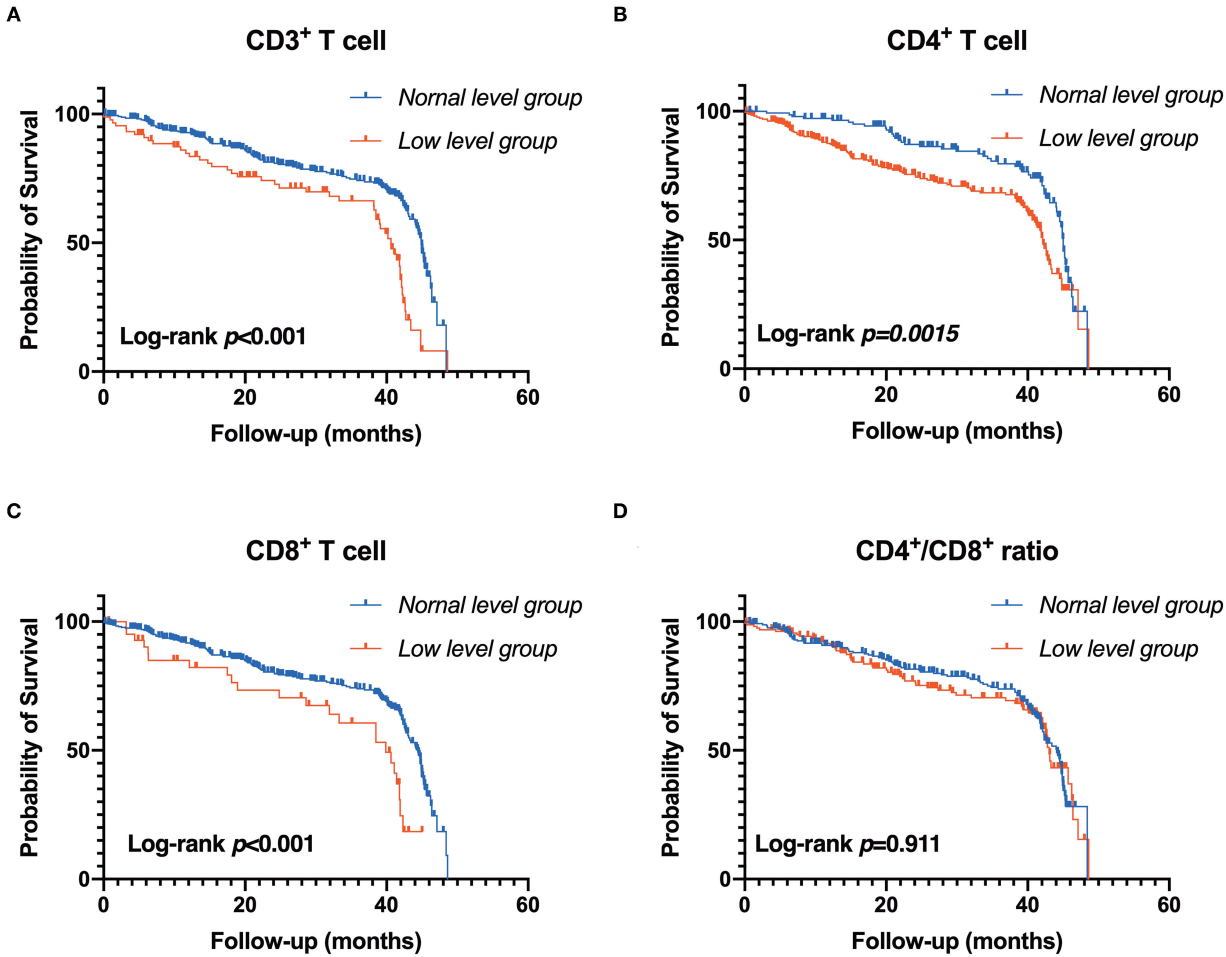
Association between T-lymphocyte subsets and renal outcome. **(A)** CD3^+^ T cell, **(B)** CD4^+^ T cell, **(C)** CD8^+^ T cell, **(D)** CD4^+^/CD8^+^ T cell. Patients were stratified into two groups by clinical reference value (Kaplan-Meier analysis with log-rank test).

During the follow-up, renal events occurred in 37.50% of patients. To evaluate the prognostic value of T lymphocytes, we categorized the patients into normal T lymphocyte and low T lymphocyte groups, and the information is presented in Table 3. According to CD3^+^ T cell level, there were significant differences in hemoglobin, iPTH, urea nitrogen, cystatin C and eGFR between the two groups, while there were no significant differences in baseline characteristics between the low level of CD4^+^ T cell group and normal level of CD4^+^ T cell group except for serum phosphorus. For CD8^+^ T cell level, there were significant differences in age, CKD stages, hemoglobin, iPTH, urea nitrogen, cystatin C and eGFR between the two groups. In addition, there was no significant difference in Erythropoietin (EPO) administration between the normal-level and the low-level T cell subpopulation (Supplementary Table 4). Moreover, Kaplan-Meier survival analysis was generated and showed that patients in low levels of CD3^+^, CD4^+^ and CD8^+^ T lymphocyte group have a higher risk of the renal outcome when compared with normal level T lymphocyte group except for CD4^+^/CD8^+^ ratio (Figure 4). Then, Cox proportional hazard model further concluded that patients had a near 2.5-fold risk of renal outcome in the low level of CD3^+^ T lymphocyte group (HR = 2.407, 95% CI: 1.664–3.482, *p* < 0.001) and low level of CD4^+^ T lymphocyte group (HR = 2.397, 95% CI: 1.633–3.518, *p* < 0.001) after adjusted by multiple models, while patients in low CD8^+^ T lymphocyte group had an approximately 1.42-fold increased risk of renal outcome after multivariable-adjusted (HR = 2.416, 95% CI: 1.476–3.955, *p* < 0.001) (Table 4; Figure 3B).

**Table 3:** Demographic, clinical and laboratory parameters in accordance with CD3^+^, CD4^+^ and CD8^+^ T cells.

**Characteristics**	**CD3** ^**+**^ **T**			**CD4** ^**+**^ **T**			**CD8** ^**+**^		
	**Normal-level group (*n* = 321)**	**Low-level group (*n* = 89)**	***P*-value**	**Normal-level group (*n* = 145)**	**Low-level group (*n* = 265)**	***P*-value**	**Normal-level group (*n* = 368)**	**Low-level group (*n* = 42)**	***P*-value**
Age, year	46.86 ± 14.20	48.67 ± 15.77	0.263	46.83 ± 13.67	47.48 ± 15.04	0.209	46.74 ± 14.43	51.71 ± 15.11	0.036
**Sex**									
Male (%)	168 (52.33)	44 (49.44)	0.628	74 (51.03)	138 (52.08)	0.840	193 (52.45)	19 (45.24)	0.376
Female (%)	153 (47.66)	45 (50.56)		71 (48.97)	127 (47.92)		175 (47.55)	23 (54.76)	
**CKD stage**									
Stage 3 (%)	158 (49.22)	29 (32.58)	0.013	61 (42.07)	126 (47.55)	0.368	177 (48.10)	10 (23.81)	0.009
Stage 4 (%)	87 (27.10)	28 (31.46)		40 (27.59)	75 (28.30)		100 (27.17)	15 (35.71)	
Stage 5 (%)	76 (23.68)	32 (35.96)		44 (30.34)	64 (24.15)		91 (24.73)	17 (40.48)	
Current smoker (%)	88 (27.41)	23 (25.84)	0.768	45 (31.03)	66 (24.91)	0.182	103 (27.99)	8 (19.05)	0.217
Hypertension, *n* (%)	136 (42.38)	29 (32.58)	0.096	56 (38.62)	109 (41.13)	0.620	151 (41.03)	14 (33.33)	0.335
Diabetes, *n* (%)	30 (9.35)	7 (7.87)	0.666	10 (6.90)	27 (10.19)	0.266	34 (9.24)	3 (7.14)	0.653
Cardiovascular disease, *n* (%)	13 (4.05)	5 (5.62)	0.523	3 (2.07)	15 (5.66)	0.090	17 (4.62)	1 (2.38)	0.502
Body-mass index, kg/m^2^	23.57 ± 3.60	23.22 ± 3.38	0.514	23.87 ± 3.67	23.29 ± 3.48	0.609	23.58 ± 3.60	22.73 ± 3.10	0.140
Systolic blood pressure, mmHg	130 (120, 140)	132 (120, 145)	0.195	131 (120, 141)	130 (120, 143)	0.599	131 (120, 145)	128 (120, 143)	0.457
Diastolic blood pressure, mmHg	80 (74, 90)	82 (75, 92)	0.392	80 (74, 90)	81 (75, 90)	0.679	81 (75, 90)	80 (70, 89)	0.123
Hemoglobin, g/L	110.45 ± 23.31	103.11 ± 25.6	0.015	109.54 ± 22.72	108.44 ± 24.50	0.180	109.89 ± 23.43	99.55 ± 25.85	0.008
Albumin, g/L	39.10 (34.90, 43.00)	39.20 (35.48, 42.23)	0.580	38.55 (34.83, 41.70)	39.50 (35.05, 43.10)	0.234	39.05 (34.98, 42.80)	39.70 (35.55, 42.15)	0.971
Phosphorus, mmol/L	1.20 (1.04, 1.39)	1.26 (1.06, 1.44)	0.261	1.27 (1.08, 1.45)	1.18 (1.02, 1.36)	0.049	1.20 (1.04, 1.39)	1.29 (1.07, 1.49)	0.179
Calcium, mmol/L	2.21 (2.09, 2.30)	2.19 (2.08, 2.29)	0.655	2.22 (2.10, 2.31)	2.19 (2.08, 2.29)	0.251	2.21 (2.09, 2.30)	2.17 (2.05, 2.32)	0.430
iPTH, pg/mL	89.20 (53.00,182.00)	138.6 (59.65, 243.25)	0.027	93.30 (51.23, 227.00)	95.20 (57.65, 193.35)	0.973	90.70 (53.15, 193.88)	144 (71.15, 303.50)	0.017
Uric acid, umol/L	467.55 ± 115.70	475.31 ± 117.73	0.947	481.53 ± 112.75	462.47 ± 117.49	0.552	468.88 ± 117.39	472.48 ± 104.82	0.849
BUN, mmol/L	10.11 (7.39, 15.09)	12.02 (8.08, 18.37)	0.044	11.30 (7.81, 17.61)	10.36 (7.40, 15.00)	0.056	10.17 (7.42, 15.90)	12.48 (10.54, 16.32)	0.041
Cystatin C, mg/L	2.40 (1.67, 3.72)	3.33 (2.21, 4.62)	<0.001	2.75 (1.78, 4.19)	2.63 (1.82, 3.93)	0.511	2.47 (1.75, 3.88)	3.68 (2.78, 4.98)	0.001
eGFR, mL/min/1.73 m^2^	29.09 (15.23, 43.93)	18.35 (11.11, 38.55)	0.004	25.20 (11.78, 43.54)	28.68 (15.14, 43.63)	0.254	28.59 (14.63, 43.84)	17.58 (9.77, 25.35)	0.003

**Table 4:** Association of CD3^+^, CD4^+^, and CD8^+^ T cells with renal outcome.

	**Model**	**HR (95% CI)**		***P***
		**Low-level group**	**Normal-level group**	
**CD3** ^**+**^				
	Model 1	2.156 (1.527–3.046)	Reference	<0.001
	Model 2	2.163 (1.523–3.072)		<0.001
	Model 3	2.097 (1.471–2.989)		<0.001
	Model 4	2.407 (1.664–3.482)		<0.001
**CD4** ^**+**^				
	Model 1	1.722 (1.226–2.419)	Reference	0.002
	Model 2	1.732 (1.226–2.446)		0.002
	Model 3	2.049 (1.429–2.937)		<0.001
	Model 4	2.397 (1.633–3.518)		<0.001
**CD8** ^**+**^				
	Model 1	2.131 (1.359–3.341)	Reference	0.001
	Model 2	2.337 (1.471–3.714)		<0.001
	Model 3	2.363 (1.457–3.834)		<0.001
	Model 4	2.416 (1.476–3.955)		<0.001

*Cox regression model for the association between CD3^+^, CD4^+^, CD8^+^ T cells level, and renal outcome*.

*Model 1: crude*;

*Model 2: age, gender, BMI, systolic blood pressure, and diastolic blood pressure adjusted*;

*Model 3: Model 2 plus CKD stage, hypertension, diabetes, cardiovascular disease, and smoker adjusted*;

*Model 4: Model 3 plus phosphorus, calcium, intact parathyroid hormone, hemoglobin, albumin, BUN, Cystatin C, and uric acid adjusted*.

## Discussion

In the current study, we found that T-lymphocyte subsets were significantly reduced in the CKD population. T-lymphocyte subsets were closely related to infection and renal outcome in patients with advanced CKD.

T-lymphocytes mainly contain CD3^+^, CD4^+^ and CD8^+^ and other members, and their expression and specific roles are quite different. For example, the CD3^+^ molecule is distributed on almost all mature T-cell surfaces and is a superficial T-lymphocyte maturation marker. The decrease of CD3^+^ T cells represents that a lower immune function of the human body (23). In a previous study, the proliferation of CD3^+^T cells was significantly decreased in ESKD patients (24). It suggested that several antibodies respond critically dependent on the presence of antigen-specific CD4^+^ T cells for its generation and maintenance. Therefore, disturbances of CD4^+^ T cells will affect cellular immunity and humoral immunity (25, 26). Additionally, CD8^+^ T cells play an essential role in anti-infective immunity, antitumor immunity, and suppression of immune system function by secreting various cytokines (27). A previous study reported that fewer circulation of CD8^+^ T cells was found in pre-dialysis patients compared to the age-matched healthy group (28). Moreover, the ratio of CD4^+^/CD8^+^ T cell is also an indicator to assess individual immune function, and this ratio indicates a balance of these functions (29). In addition, a small number, the cross-sectional study performed by Hartzell et al. showed that lower level of CD3^+^ T cells in CKD and lower level of CD8^+^ and CD4^+^/CD8^+^ T cell in ESKD patients. In contrast, no difference was noticed in total T cells among healthy controls, CKD, and ESKD patients. Consequently, they also analyzed the relation between exhausted T cell phenotype and CKD. And they concluded that these alterations were companied by a more pro-inflammatory phenotype in kidney failure patients (13). Our study further strengthened their results with an extended number of participants in CKD while not in dialysis patients. Moreover, we also investigated the T-lymphocyte subsets and infection and renal outcome. In addition, we further noticed that the level of T-lymphocyte subsets is associated with CKD staging. Suggesting that, with the kidney function deterioration, T-lymphocyte subsets will be further reduced. These results indicated that different phenotypes of T cells might play different roles in the CKD niche and adverse outcomes. The dysfunction of T cell-mediated immune responses is observed in CKD patients with immunodeficiency (30). Previous studies found a low T-lymphocyte function in CKD patients, and T-cell subgroup detection, an indicator of cellular immune function, was often abnormal (31). For example, patients with IgA glomerulonephritis nephropathy (IgAN) showed lower activated T-lymphocytes in peripheral blood than healthy people (32). Moreover, protein-energy malnutrition (PEM), a common and severe complication of CKD, induces T-lymphopenia and T-lymphocyte dysfunction (33). Several studies indicated that ESKD patients have T-lymphocyte dysfunctions, such as the reduction of regulatory T-cells (Tregs), a subgroup of CD4^+^ T cells (34, 35). Furthermore, because Tregs inhibit systemic inflammation and CVD, deficiency of Tregs might be one reason for some complications in ESRD patients, including CVD (36). The changes of T-lymphocyte subpopulations also accelerate the progression of DN (15). Currently, minimal change disease (MCD) is believed to be due to podocyte damaged by glomerular permeability factors produced from abnormal T-lymphocyte subsets (37, 38). Another study found that CD4^+^ T cells in patients with relapsed MCD were significantly upregulated (39), further confirming the role of T cells in the development of this condition. Thus, the occurrence of kidney diseases is related to the immune environment, especially the number and function of T-lymphocytes. Therefore, it is necessary to elucidate the role of T- lymphocytes in the progression of CKD.

In the current study, we added risk factors related to CKD and cellular immune function for correction. For instance, a previous study reported that old age leads to immunosenescence, with a decrease in T cells in the process of aging (40). Furthermore, most likely, owing to different lifestyles in Western and Asian countries, accumulated risk factors such as hypertension, glucose intolerance, obesity, and hypercholesterolemia enhance the prevalence of CKD over time (41). Therefore, our study included hypertension, diabetes, and BMI as risk factors for adjustment. Other traditional risk factors for the renal outcome, such as hemoglobin, albumin, phosphorus, calcium, iPTH, uric acid, BUN, cystatin, and eGFR, are also taken into account. After correction, groups with low-level of CD3^+^, CD4^+^, and CD8^+^ T cells subsets showed a significantly increased risk of renal outcomes and infection. These results indicate that T cells subsets can be used as prognosis biomarkers in patients with CKD. Furthermore, surveillance of the markers routinely can be beneficial to patients with CKD.

A previous study demonstrated that low levels of T-lymphocytes (mainly CD4^+^ and CD8^+^ T cells) are associated with an inadequate response to EPO. More pro-inflammatory factors such as interleukin-4 (IL-4), IL-10, and tumor necrosis factor-α (TNF -α) were secreted in patients with a low circulation of T-lymphocytes (42). Recent evidence shows that EPO has a vital role in immune-modulating effects through the ERP receptor in various organs. EPO can also inhibit human T cells *in vivo* study (43). Donadei et al. demonstrated that EPO overexpression or recombinant EPO (rEPO) management restricts the formation of Th17 cells, which was produced by CD4^+^ cells (44), indicating that EPO has a close relationship with T-lymphocyte subsets. In our study, a low level of hemoglobin was in company with a low level of CD3^+^ and CD8^+^ T cells (Table 3). On the one hand, we spectated that anemia in CKD pre-dialyzed patients might be attributed to abnormal immune function. On the other hand, the EPO deficiency in CKD patients may also contribute to the dysfunction of T-lymphocyte subsets. There were no significant differences in EPO administration between the normal-level and the low-level T cell subpopulation. Unfortunately, we didn't measure and compared the EPO levels in these CKD patients between normal and low-level T cell groups. Hence, but the specific mechanism is still unclear, further studies are urgently needed. Bouts et al. reported that total T-lymphocytes and T-lymphocyte subsets were decreased in children with renal failure regardless of dialysis, compared with those in the healthy group (45). Although low levels of CD3^+^, CD4^+^, and CD8^+^ T cells were identified as significant risk factors for CKD progression, we only focused on the baseline levels of T-lymphocytes and did not continue to track changes after dialysis. Borges et al. showed that patients with ESKD had reduced levels of circulating CD3^+^ T cells (46). Although the level of circulating CD3^+^ T cells has a downtrend in CKD stage 5 compared with CKD stages 3–4, no significant difference was found in our study. This finding might be due to the small sample size. A study stated that T-lymphocytes and their subsets were not associated with eGFR (47). However, our study showed that CD3^+^ and CD8^+^ T cells were associated with eGFR, but CD4^+^ T cells were significantly correlated with eGFR. One advantage of our study is the emphasis on the relationship between pre-dialysis immune function and renal outcome, which has not been frequently examined in previous studies, and early attention to these factors can help to guide treatment. Nevertheless, a limitation of this study is the fact that we did not examine changes in the phenotype and function of immune cells.

In conclusion, our results reveal those low levels of CD3^+^, CD4^+^, and CD8^+^ T cells subsets are associated with an increased risk of infection and renal outcome in pre-dialysis CKD stage 3–5 patients, which indicates that T-lymphocyte subsets might be used as a predictor factor of infection and renal outcome in those patients.

## Data Availability Statement

The original contributions presented in the study are included in the article/Supplementary Material, further inquiries can be directed to the corresponding author/s.

## Ethics Statement

The studies involving human participants were reviewed and approved by the Ethical committee of Xinqiao Hospital. The patients/participants provided their written informed consent to participate in this study. Written informed consent was obtained from the individual(s) for the publication of any potentially identifiable images or data included in this article.

## Author Contributions

JX and YQ drafted the manuscript, as well as the collection, acquisition, analysis, and interpretation of data. ZY analyzed the data. TH and JZ contributed to the conception and design of the current study. YH and KY participated in the coordination of the study and reviewed the manuscript. JZ designed and supervised this study, analyzed data, wrote, and revised the manuscript. All authors contributed to the article and approved the submitted version.

## Funding

This study was supported by the Personal Training Program for Clinical Medicine Research of Army Medical University (2018XLC1007) and Frontier-specific projects of Xinqiao Hospital (2018YQYLY004).

## Conflict of Interest

The authors declare that the research was conducted in the absence of any commercial or financial relationships that could be construed as a potential conflict of interest.

## Publisher's Note

All claims expressed in this article are solely those of the authors and do not necessarily represent those of their affiliated organizations, or those of the publisher, the editors and the reviewers. Any product that may be evaluated in this article, or claim that may be made by its manufacturer, is not guaranteed or endorsed by the publisher.

## Supplementary Material

Supplementary Figure 1 The flowchart of the study.

Supplementary Figure 2 Frequency distribution of T-lymphocyte subsets in CKD patients. **(A)** CD3^+^ T cell, **(B)** CD4^+^ T cell, **(C)** CD8^+^ T cell.

Supplementary Table 1 The relationship between the T lymphocyte subsets in CKD patients and healthy control.

Supplementary Table 2 The basic characteristics of the included CKD patients according to infection.

Supplementary Table 3 Infection of normal and low level T lymphocyte subsets.

Supplementary Table 4 Proportion of erythropoietin administration in accordance with CD3^+^, CD4^+^ and CD8^+^ T cells.
