# Supplementary figures and images for: T-Lymphocyte Subsets Alteration, Infection and Renal Outcome in Advanced Chronic Kidney Disease
Source: Front Med (Lausanne). 2021 Sep 9;8:742419. doi: 10.3389/fmed.2021.742419 (PMC8458643; doi:10.3389/fmed.2021.742419)

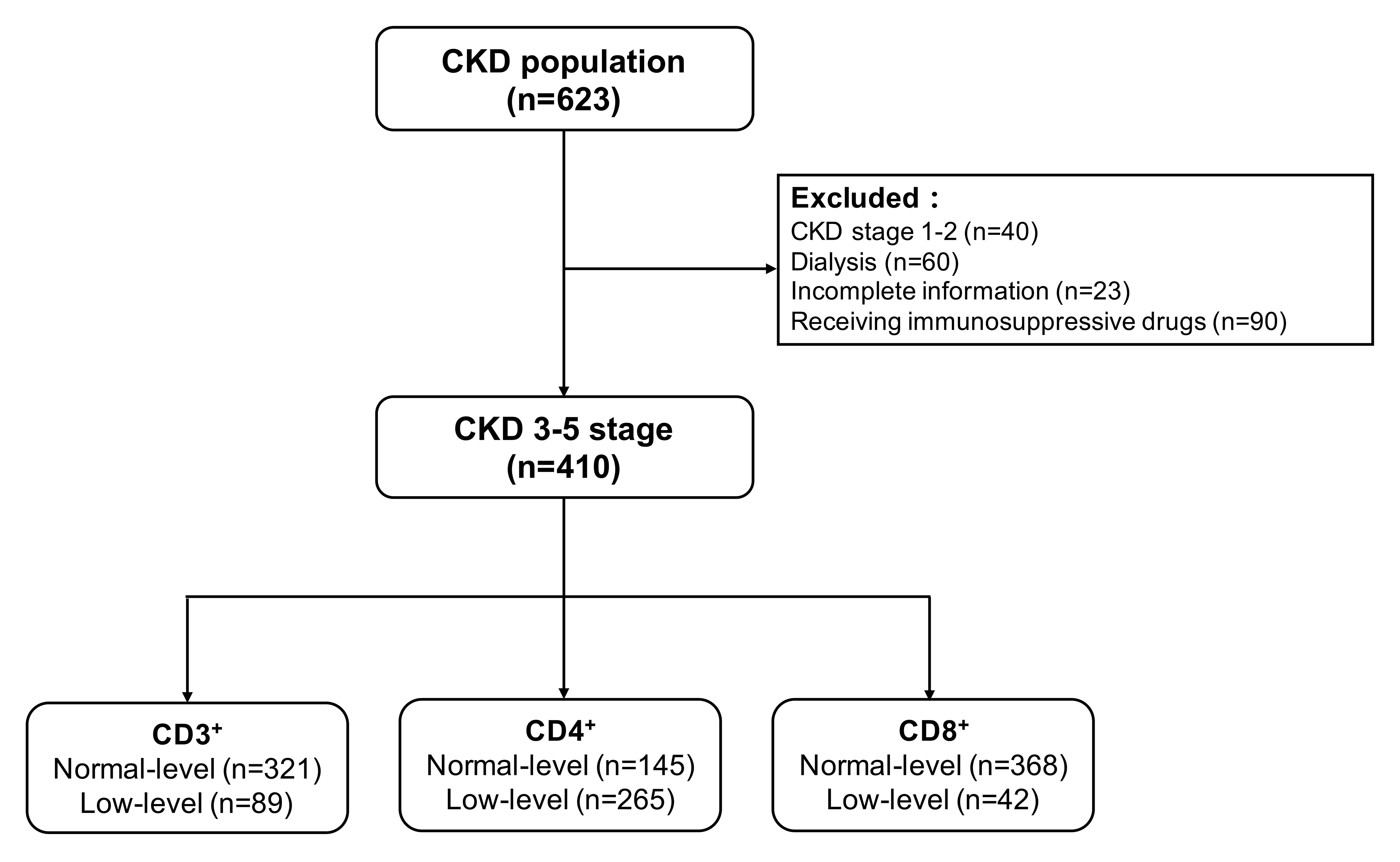

Supplement: Supplementary Figure 1 — The flowchart of the study. [file Image_1.TIF]

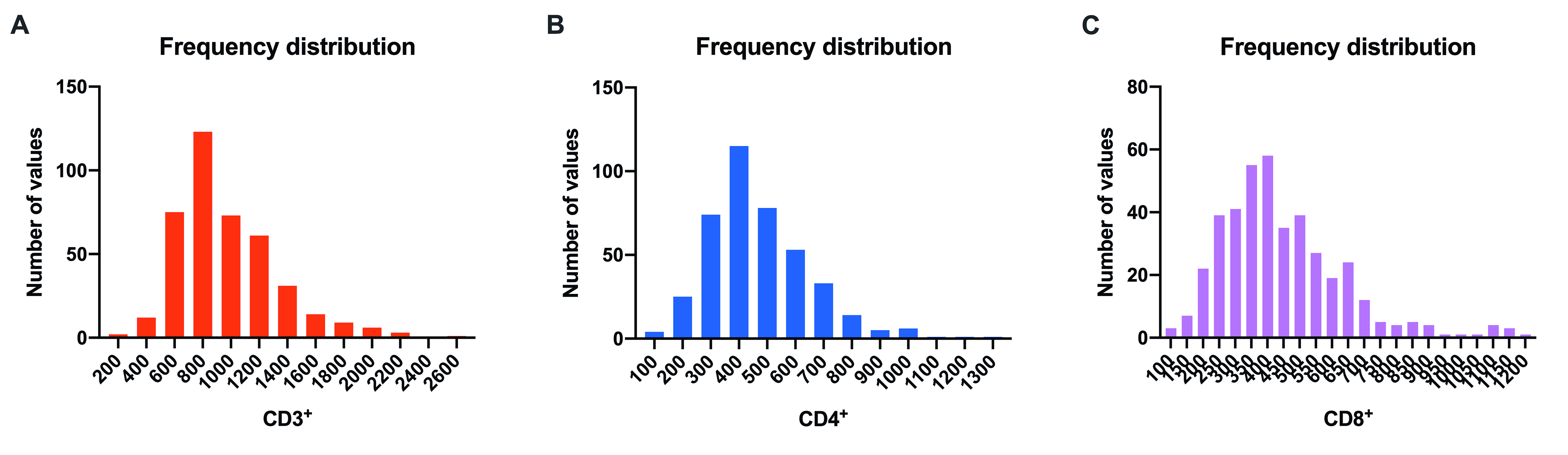

Supplement: Supplementary Figure 2 — Frequency distribution of T-lymphocyte subsets in CKD patients. (A) CD3+ T cell, (B) CD4+ T cell, (C) CD8+ T cell. [file Image_2.TIFF]
