# Supplementary material for: T-Lymphocyte Subsets Alteration, Infection and Renal Outcome in Advanced Chronic Kidney Disease
Source: Front Med (Lausanne). 2021 Sep 9;8:742419. doi: 10.3389/fmed.2021.742419 (PMC8458643; doi:10.3389/fmed.2021.742419)
Supplement: Supplementary Table 1 — The relationship between the T lymphocyte subsets in CKD patients and healthy control. [file Data_Sheet_1.docx]

Supplementary Material

| **Supplementary Table 1. The relationship between the T lymphocyte subsets in CKD patients and healthy control** | | | | | |
| --- | --- | --- | --- | --- | --- |
| **T lymphocyte subsets** | **Healthy control**  **(n=100)** | **CKD 3**  **(n=187)** | **CKD 4**  **(n=115)** | **CKD 5**  **(n=108)** | ***P*** |
| CD3^+^ | 1655 (925，2175) | 902 (769，1172) | 972 (716，1268) | 896 (704，1232) | <0.001 |
| CD4^+^ | 944 (764，1099) | 444 (352，571) | 444 (344，640) | 468 (340，630) | <0.001 |
| CD8^+^ | 728 (465，940) | 420 (336，551) | 440 (328，612) | 400 (302，512) | <0.001 |
| CD4^+^/CD8^+^ | 1.57 (0.96，1.94) | 1.00 (0.90，1.30) | 1.00 (0.80，1.40) | 1.20 (0.90，1.56) | 0.025 |

| **Supplementary Table 2. The basic characteristics of the included CKD patients according to infection** | | | | |
| --- | --- | --- | --- | --- |
|  | **Infection group**  **(n = 62)** | **Non-infection group**  **(n =348)** | **t/z/χ2** | ***P*** |
| CKD stage |  |  |  |  |
| CKD3(%) | 19(30.65) | 168(48.28) | 7.256 | 0.027 |
| CKD4(%) | 20(32.26) | 95(27.30) |  |  |
| CKD5(%) | 23(37.10) | 85(24.43) |  |  |
| Age, years | 48.53 $\pm$ 15.76 | 47.02 $\pm$ 14.34 | 0.752 | 0.453 |
| Female, n (%) | 27(43.55) | 171(49.14) | 0.658 | 0.417 |
| Current smoker, n (%) | 22(35.48) | 89(25.57) | 2.617 | 0.106 |
| Hypertension, n (%) | 20(32.26) | 145(41.67) | 1.937 | 0.164 |
| Diabetes, n (%) | 2(3.23) | 35(10.06) | 2.992 | 0.084 |
| Cardiovascular disease, n (%) | 1(1.61) | 17(4.89) | 1.342 | 0.247 |
| Body-mass index, kg/m^2^ | 23.67 $\pm$ 3.34 | 23.46 $\pm$ 3.60 | 0.415 | 0.678 |
| Systolic blood pressure, mmHg | 127(119，150) | 130(120，142) | -0.080 | 0.936 |
| Diastolic blood pressure, mmHg | 80(70，91.5) | 81(75，90) | -0.689 | 0.491 |
| Hemoglobin, g/L | 105.13 $\pm$ 21.71 | 109.49 $\pm$ 24.20 | -1.326 | 0.186 |
| Albumin, g/L | 39.15(35.10，42.80) | 39.50(34.00，42.45) | -0.911 | 0.362 |
| Phosphorus, mmol/L | 1.25(1.07，1.47) | 1.20(1.04，1.38) | -1.192 | 0.233 |
| Calcium, mmol/L | 2.14(2.02，2.29) | 2.21(2.09，2.30) | -1.392 | 0.164 |
| iPTH, pg/mL | 116(48.35，269.6) | 92.70(56.13，196.63) | -0.567 | 0.571 |
| Creatine, μmol/L | 257(168.75，416.45) | 207.95(141.95，333.75) | -2.064 | 0.039 |
| Uric acid, μmol/L | 502.70 $\pm$ 127.32 | 463.25 $\pm$ 113.06 | 2.480 | 0.014 |
| BUN, mmol/L | 13.57(9.51，19.26) | 10.23(7.38，14.98) | -2.822 | 0.005 |
| eGFR, mL/min/1.73m^2^ | 19.40(10.15，31.19) | 28.46(14.86，44.00) | -3.283 | 0.001 |
| Cystatin C, mg/L | 3.22(2.37，4.58) | 2.48(1.72，3.80) | -3.195 | 0.001 |

| **Supplementary Table 3. Infection of normal and low level T lymphocyte subsets** | | | |
| --- | --- | --- | --- |
| **T lymphocyte subsets** | **Number of infection(%)** | **χ^2^** | ***P*** |
| **CD3^+^ T** |  |  |  |
| Normal-level group (n=321) | 40(12.46) | 8.157 | 0.004 |
| Low-level group (n=89) | 22(24.72) |  |  |
| **CD4^+^ T** |  |  |  |
| Normal-level group (n=145) | 12(8.28) | 8.192 | 0.004 |
| Low-level group (n=265) | 50(18.87) |  |  |
| **CD8^+^ T** |  |  |  |
| Normal-level group (n=368) | 59(16.03) | 2.321 | 0.128 |
| Low-level group (n=42) | 3(7.14) |  |  |
| **CD4^+^/CD8^+^** |  |  |  |
| Normal-level group (n=249) | 22(8.84) | 19.525 | <0.001 |
| Low-level group (n=161) | 40(24.84) |  |  |

| **Supplementary Table 4. Proportion of erythropoietin administration in accordance with CD3^+^，CD4^+^ and CD8^+^ T cells** | | | | | | | | |
| --- | --- | --- | --- | --- | --- | --- | --- | --- |
| **CD3^+^ T** | | | **CD4^+^ T** | | | **CD8^+^** | | |
| **Normal-level group (n=321)** | **Low-level group (n=89)** | **P value** | **Normal-level group (n=145)** | **Low-level group (n=265)** | **P value** | **Normal-level group (n=368)** | **Low-level group (n=42)** | **P value** |
| 100(31.15%) | 36(40.45%) | 0.099 | 49(33.79%) | 87(32.83%) | 0.843 | 118(32.07%) | 18(42.86%) | 0.159 |
